# Comparing the real-world effectiveness of botulinum toxin type A injections across distinct poststroke muscle hyper-resistance patterns

**DOI:** 10.3389/fneur.2026.1836266

**Published:** 2026-07-02

**Authors:** Xuncan Liu, Chen Chen, Yanmin Ju, Liang Zhao, Guoxing Xu, Yinxing Cui, He Li, Xiaowei Chen, Zhenlan Li

**Affiliations:** 1 Department of Rehabilitation, The First Hospital of Jilin University, Changchun, Jilin, China; 2 Department of Pediatric Neurology, The First Hospital of Jilin University, Changchun, Jilin, China

**Keywords:** BoNT-A, contracture, hyper-resistance, spasticity, stroke

## Abstract

**Background:**

Post-stroke muscle hyper-resistance is produced by both neurogenic (spasticity) and non-neurogenic (contracture) factors. BoNT-A is the most effective intervention for post-stroke spasticity, yet whether concomitant contracture alters its therapeutic benefit remains unclear.

**Aims:**

To compare BoNT-A effectiveness in plantar-flexor hyper-resistance stratified by contracture.

**Methods:**

We retrospectively reviewed stroke survivors with spastic hemiplegia and ankle plantar-flexor hyper-resistance who received BoNT-A injections. Patients were stratified into two groups according to the presence of restricted passive ankle dorsiflexion: the spasticity group (PROM limitation <7°) and the spasticity-with-contracture group (PROM limitation ≥7°). Outcomes were assessed at baseline and at 2, 4 and 12 weeks post-injection, including the Modified Ashworth Scale (MAS) for plantar-flexors, Brunnstrom Recovery Stage (BRS), Fugl–Meyer Assessment (FMA) lower-extremity subscore and Barthel Index (BI).

**Results:**

A total of 107 patients were enrolled—54 in the spasticity group and 53 in the spasticity-with-contracture group. Baseline comparison revealed a significantly longer disease duration in the spasticity-with-contracture group; other characteristics were comparable. Both groups achieved improvements in MAS and BRS at all three follow-up visits. FMA and BI improved in the spasticity group at 4 and 12 weeks, whereas the spasticity-with-contracture group showed improvement only at 12 weeks. Between-group analyses indicated that MAS and BRS scores were consistently better in the spasticity group at each time point; although median FMA and BI were numerically higher in this group, the differences did not reach statistical significance.

**Conclusion:**

BoNT-A markedly reduces post-stroke hyper-resistance and enhances motor function and activities of daily living; by contrast, concomitant contracture is associated with delayed and attenuated improvement in MAS and BRS.

## Introduction

Spasticity after stroke is a common sensorimotor control disorder, with an incidence rate of 17 to 42.6% being observed among stroke patients (1). If spasticity is not promptly and effectively treated, it can lead to soft tissue contractures, thereby potentially resulting in the complete loss of motor function. Spasticity, whether occurring alone or with contracture, not only compromises motor recovery after stroke but also induces pain, heightens caregiver burden, and increases the risk of depression—consequently prolonging rehabilitation periods and substantially raising medical costs (2–4). Therefore, the prevention and treatment of spasticity/contracture after stroke has become an urgent problem in the field of rehabilitation medicine.

The standard clinical assessment for spasticity and contracture involves passively stretching the affected muscle to determine the onset and quality of resistance (5, 6). Both conditions present with increased resistance to passive movement. However, accumulating evidence indicates that the Modified Ashworth Scale (MAS) cannot differentiate between these two distinct pathological entities (3, 7). Consequently, in clinical practice they are frequently confused or described by interchangeable terms such as hypertonia, stiffness, or hypoextensibility (7, 8). Therefore, the European consensus has standardised the terminology for this phenomenon, adoptingthe term “hyper-resistance” to describe impaired neuromuscular response during passive stretching (9). In this study, “spasticity” is defined according to Lance (10) as a motor disorder characterised by a velocity-dependent increase in tonic stretch reflexes with exaggerated deep tendon reflexes, and as a positive manifestation of the upper motor neuron syndrome. This constitutes the neurogenic component of hyper-resistance (9). “Contracture” refers to increased resistance arising from non-neurogenic factors, including structural changes in soft tissues such as muscles, tendons, fascia, joint capsules, and associated connective tissues (11). This represents the non-neurogenic component of hyper-resistance (9). Therefore, hyper-resistance encompasses both spasticity and contracture, and the two conditions frequently coexist and are readily conflated in clinical practice. Currently, there is no universally agreed diagnostic criterion for contracture in clinical practice, and different studies have adopted varying standards, namely the minimal important change (MIC) and minimal clinically important difference (MCID) in passive range of motion (PROM) across joints (12). Mehrholz and colleagues proposed a 10° change in PROM as the MCID (13), whereas a recent Cochrane systematic review advocated a 10% change relative to the normal PROM as the MCID for contracture assessment (11). In the present study, we adopted this 10% threshold as the criterion for contracture, with the reference range for passive ankle motion being 70° (45° plantarflexion and 25° dorsiflexion), thereby defining contracture as a PROM limitation of ≥7°.

Previous clinical studies have mainly focused on observing the efficacy of BoNT-A in terms of treating spasticity after stroke (14, 15). Although BoNT-A can reduce the tension of spastic muscles, its effects on patients’ motor function, daily living ability, and gait are quite different. One possible reason for these findings is that the included research subjects were not consistent, and most of the studies did not distinguish whether the hyper-resistance of the muscles involved simple spasticity or spasticity accompanied by contracture. Therefore, we designed this retrospective clinical study to observe the clinical efficacy of BoNT-A injections in different types of post-stroke hyper-resistance in the ankle plantar-flexor muscles.

## Methods

### Study design and subjects

This study was a retrospective, observational clinical study. Patients with poststroke spastic hemiplegia who were hospitalised in the Rehabilitation Department of the First Hospital of Jilin University from April 2017 to April 2024 were included. These patients demonstrated hyper-resistance in the plantar flexor muscles of their ankle joints and received BoNT-A (Hengli^®^, Lanzhou Biological Products Institute, Lanzhou, China) injection treatment under ultrasonographic guidance. Targeted muscles included the gastrocnemius, soleus, tibialis posterior, as well as the flexor digitorum longus and flexor hallucis longus muscles. The toxin was diluted to a concentration of 3 mL of normal saline per 100 units, with a total dose ranging from 200 to 400 units. Hyper-resistance was operationally defined as MAS grade ≥1 + in the ankle plantar flexors. This threshold was selected as it represents the minimum grade at which BoNT-A intervention is clinically indicated in our institution. All patients received conventional rehabilitation treatment programs, including physical therapy, occupational therapy, assistive device training, physical modalities and traditional Chinese medicine. The treatment was administered once a day, 6 days per week. The inclusion and exclusion criteria for the study subjects were as follows.

#### Inclusion criteria

1) Ages between 14 and 75 years, regardless of sex;
2) Meeting the diagnostic criteria for stroke and the occurrence of stroke representing the first onset;
3) With hemiplegia and ankle plantar flexor hyper-resistance of MAS grade ≥ 1 + on the affected side;
4) Patients receiving first-time BoNT-A injections for lower-limb hyper-resistance;
5) No severe cognitive impairment, with rehabilitation assessments being regularly performed before and after the BoNT-A injections.

#### Exclusion criteria

1) Previous history of stroke;
2) Complicated with other cranial or spinal diseases causing paralysis and hyper-resistance, such as intracranial space-occupying lesions, intracranial infectious lesions, and spinal cord injuries;
3) Patients with previous lower limb disabilities due to musculoskeletal system diseases;
4) Recurrence of stroke during the study period;
5) Previous BoNT-A injections to the lower limbs, especially the ankle plantar flexor muscles (gastrocnemius, soleus, tibialis posterior, flexor digitorum longus, or flexor hallucis longus). Patients with BoNT-A injections to other body regions (e.g., upper limb) were not excluded if the ankle plantar flexors were naive to treatment.;
6) Severe cognitive impairment affecting the assessment results;
7) Missing data or incomplete rehabilitation assessment results for various reasons;
8) Other situations deemed to be unsuitable for inclusion by the researchers.

### Study procedure

The patients were divided into a spasticity group and a spasticity-with-contracture group based on whether they demonstrated a limited PROM of the ankle joint dorsiflexion before the injection treatment (PROM limitation ≥ 7°). Follow-up was conducted at 2 weeks, 4 weeks, and 12 weeks after BoNT-A injection. The collected baseline and follow-up data included demographic information (including sex, age, height, and weight), stroke type, medical history, past medical history, ankle joint PROM, MAS for assessing ankle plantar flexion, the Brunnstrom staging (BRS) system and Fugl-Meyer assessment (FMA) for assessing the affected lower limb, and the Barthel Index (BI) for assessing activities of daily living. The study protocol was reviewed and approved by the Ethics Committee of the First Hospital of Jilin University (ethics number: 2024-1095).

### Statistical analysis

The data were analysed using SPSS 23.0 statistical software (IBM Company, Armonk, New York, United States). The Kolmogorov–Smirnov test was used to assess the normal distribution of the continuous variables. Quantitative data that satisfied the normal distribution criteria are described as x̄ ± s. Data that satisfied the normal distribution criteria and were homogeneous in variance were compared within groups using paired-sample t tests and between groups using two independent-sample t tests. Quantitative data that did not meet the assumption of normality are presented as medians with interquartile ranges (25th and 75th percentiles) [M(P25, P75)]. Within-group comparisons before and after the treatment were performed via the Wilcoxon signed-rank test, and between-group comparisons were performed via the Mann–Whitney test. Count data are expressed as frequencies and percentages (%). Comparisons were performed via the chi-square test. A two-sided *p* < 0.05 was considered to be statistically significant.

To evaluate the dynamic impact of the treatment intervention on the indicators, this study compared the data of the same subject at 4 time points (including at baseline, the 2nd week, the 4th week, and the 12th week) via the nonparametric Wilcoxon signed-rank test, which did not require the data to follow a normal distribution. Given that this study involved 6 independent pairwise comparisons across 4 time points, there was a risk of Type I error (false positive) inflation due to the use of multiple comparisons. To control the familywise error rate, this study applied the Bonferroni correction scheme, adjusting the significance level to *α* = 0.05/6 ≈ 0.0083.

## Results

### Patient demographics and disease characteristics

A total of 107 patients were included in this study, of whom 54 were assigned to the spasticity group and 53 to the spasticity-with-contracture group.

The spasticity group comprised 33 men (61.11%) and 21 women (38.89%), with a mean age of 48.17 ± 12.84 years; the spasticity-with-contracture group comprised 37 men (69.81%) and 16 women (30.19%), with a mean age of 50.06 ± 14.04 years. In the spasticity group, 40 patients (74.07%) had a disease duration of ≤3 months, 9 (16.67%) had 3–6 months, and 5 (9.26%) had ≥6 months. In the spasticity-with-contracture group, 16 (30.19%) had ≤3 months, 20 (37.74%) had 3–6 months, and 17 (32.08%) had ≥6 months.

Comparison of baseline characteristics between groups revealed a statistically significant difference in disease duration (*p* < 0.001), whereas age, sex, BMI, stroke type, history of diabetes, and prevalence of hypertension did not differ significantly (*p* > 0.05). No significant between-group differences were observed in baseline MAS scores of the affected ankle plantar flexors, BRS, FMA-lower extremity, or Barthel Index (*p* > 0.05). These data are summarised in Table 1.

**Table 1:** Baseline demographics and disease characteristics in both groups.

Characteristic	Spasticity (54)	Spasticity with contracture (53)	*p*
Gender, *n* (%)			0.344
Female	21 (38.89)	16 (30.19)	
Male	33 (61.11)	37 (69.81)	
Age, yr. (x̄ ± s)	48.17 ± 12.84	50.06 ± 14.04	0.473
Duration, m (%)			**<0.001**
≤3 m	40 (74.07)	16 (30.19)	
3–6 m	9 (16.67)	20 (37.74)	
≥6 m	5 (9.26)	17 (32.08)	
BMI (x̄ ± s)	25.48 ± 3.43	24.73 ± 3.36	0.259
Stroke type, *n* (%)			0.288
Haemorrhagic	20 (37.04)	25 (47.17)	
Ischaemic	34 (62.96)	28 (52.83)	
Diabetes (%)	16 (29.63)	15 (28.30)	0.880
Hypertension (%)	31 (57.41)	31 (58.49)	0.910

Bold values indicate statistically significant results (*p* < 0.05).

### BoNT-A efficacy comparison for different types of hyper-resistance

#### Comparison of MAS improvement between groups

At 2, 4, and 12 weeks after treatment, MAS values in both groups differed significantly from baseline (*p* < 0.001) (see Table 2), indicating that BoNT-A reduced hyper-resistance regardless of whether contracture was present. In the spasticity group, no statistically significant differences were observed in MAS scores between 2 and 4 weeks, 2 and 12 weeks, or 4 and 12 weeks after treatment (*p* > 0.0083), suggesting that BoNT-A achieved its maximum therapeutic effect within 2 weeks and this effect persisted for up to 12 weeks. In the spasticity-with-contracture group, a statistically significant difference was observed in MAS scores between 2 and 4 weeks (*p* = 0.004), whereas no statistically significant differences were observed between 2 and 12 weeks or between 4 and 12 weeks (*p* = 0.129 and *p* = 0.180, respectively), indicating that the improvement in passive resistance was greater at 4 weeks than at 2 weeks.

**Table 2:** Pairwise comparison results between the MAS scores within each group before and after BoNT-A injection.

Group	Baseline/week 2 (*p*)	Baseline/week 4 (*p*)	Baseline/week 12 (*p)*	Week 2/week 4 (*p*)	Week 2/week 12 (*p*)	Week 4/week 12 (*p*)
Spasticity	**0.001**	**0.001**	**0.001**	0.099	0.187	0.895
Spasticity with contracture	**0.003**	**0.001**	**0.001**	**0.004**	0.129	0.180

After correction for the significance level, α = 0.05/6 ≈ 0.0083, bold values indicate statistically significant results (*p* < 0.0083).

Changes in MAS scores between groups following BoNT-A treatment are summarised in Table 3. Statistically significant differences were observed at all three follow-up time points (*p* < 0.05). These findings indicate that the reduction in MAS scores was less pronounced in the spasticity-with-contracture group compared with the spasticity group.

**Table 3:** Changes in the MAS scores after BoNT-A injection in both groups [M (P25, P75)].

Group	Week 2	Week 4	Week 12
Spasticity	1.50 (1.00, 1.50)	1.00 (1.00, 1.50)	1.00 (1.00, 1.50)
Spasticity with contracture	1.50 (1.50, 2.50)	1.50 (1.00, 1.50)	1.50 (1.50, 2.00)
*p* value	**0.002**	**0.006**	**0.001**

Bold values indicate statistically significant results (*p* < 0.05).

#### Comparison of motor function improvement between groups

Changes in lower-limb BRS scores following BoNT-A injection are summarised in Table 4. Significant improvements from baseline were observed in both groups at 2, 4, and 12 weeks (*p* < 0.0083). Significant differences were also noted between 2 and 4 weeks, and between 2 and 12 weeks; however, no significant difference was found between 4 and 12 weeks (*p* > 0.0083). These findings suggest that lower-limb motor function recovered progressively following BoNT-A injection, with improvement plateauing after 4 weeks.

**Table 4:** Pairwise comparison results between the BRS scores within each group before and after BoNT-A injection.

Group	Baseline/week 2 (*p*)	Baseline/week 4 (*p*)	Baseline/week 12 (*p*)	Week 2/week 4 (*p*)	Week 2/week 12 (*p*)	Week 4/week 12 (*p*)
Spasticity	**0.001**	**0.001**	**0.001**	**0.003**	**0.004**	0.660
Spasticity with contracture	**0.001**	**0.001**	**0.001**	**0.001**	**0.001**	0.798

After correction for the significance level, α = 0.05/6 ≈ 0.0083, bold values indicate statistically significant results (*p* < 0.0083).

Between-group comparison revealed statistically significant differences at all follow-up time points (*p* < 0.05); these data are presented in Table 5. These findings suggest that lower-limb motor function recovery following BoNT-A treatment was less pronounced in the spasticity-with-contracture group than in the spasticity group.

**Table 5:** Changes in BRS scores after BoNT-A injection in both groups [M (P25, P75)].

Group	Week 2	Week 4	Week 12
Spasticity	4.00 (3.75, 5.00)	5.00 (4.00, 5.00)	5.00 (4.00, 5.00)
Spasticity with contracture	3.00 (3.00, 4.00)	4.00 (3.00, 5.00)	4.00 (4.00, 4.50)
*p* value	**<0.001**	**0.009**	**0.003**

Bold values indicate statistically significant results (*p* < 0.05).

Changes in lower-limb FMA scores over time are summarised in Table 6. In the spasticity group, scores differed significantly from the baseline at 4 and 12 weeks, with a further significant difference observed between 2 and 12 weeks. These findings suggest that lower-limb motor function had begun to improve by week 4, with the most pronounced improvement at week 12. In the spasticity-with-contracture group, a significant difference was observed only between baseline and 12 weeks; no significant differences were found at other times. This indicates that BoNT-A-induced motor function improvement was first evident at week 12, suggesting that motor function recovery was delayed in patients with co-existing spasticity and contracture.

**Table 6:** Pairwise comparison results regarding the FMA scores within each group before and after BoNT-A injection.

Group	Baseline/week 2 (*p*)	Baseline/week 4 (*p*)	Baseline/week 12 (*p*)	Week 2/week 4 (*p*)	Week 2/week 12 (*p*)	Week 4/week 12 (*p*)
Spasticity	0.076	**0.003**	**0.001**	0.190	**0.002**	0.036
Spasticity with contracture	0.065	0.104	**0.001**	0.792	0.067	0.138

After correction for the significance level, α = 0.05/6 ≈ 0.0083, bold values indicate statistically significant results (*p* < 0.0083).

Between-group comparison of FMA scores revealed that, although the median score in the spasticity group was consistently greater than that in the spasticity-with-contracture group at all follow-up time points, these differences did not reach statistical significance differences (*p* > 0.05).

#### Comparison of BI changes between groups

Within-group BI changes showed that in the spasticity group, scores improved significantly from baseline at both 4 and 12 weeks post-injection (*p* < 0.0083), with no significant change at 2 weeks. These findings suggest that patients in this group experienced meaningful activities of daily living gains by week 4, with benefits sustained through week 12. In Contrast, in the spasticity-with-contracture group, BI improved significantly from baseline only at 12 weeks (*p* < 0.0083), indicating that BoNT-A produced delayed but clinically relevant activities of daily living improvements in this cohort.

Between-group comparison of BI changes revealed that, although median BI scores were consistently higher in the spasticity group than in the spasticity-with-contracture group at all follow-up time points, no significant between-group differences were observed (*p* > 0.05).

## Discussion

The main finding of this study is that BoNT-A effectively ameliorates the severity of post-stroke hyper-resistance, whilst enhancing motor function and activities of daily living. Significant improvements in MAS and BRS were observed as early as 2 weeks following BoNT-A injection; however, amelioration of FMA-lower extremity and BI was not evident until 4 weeks or 12 weeks. Furthermore, the co-occurrence of contracture with BoNT-A treatment is associated with diminished therapeutic outcomes, particularly regarding MAS and BRS scores.

In the group with both spasticity and contracture, the improvement of MAS by BoNT-A was less than that in the group with simple spasticity, and the optimal therapeutic effect occurred later, at 4 weeks. For patients with both spasticity and contracture, the muscle-tendon units were in a shortened state. At 2 weeks, BoNT-A could have a good blocking effect on the neuromuscular junction, and the resistance from the neurogenic component had improved, but the resistance from the non-neurogenic component had not been well relieved, resulting in less improvement of MAS than the group with simple spasticity. Additionally, after all patients received BoNT-A injection, they all routinely received traditional rehabilitation treatment, which would alleviate the non-neurogenic contribution to increased resistance and thus accounts for the delayed peak response in the spasticity-with-contracture group until week 4. The improvement in lower-limb motor function following stroke with BoNT-A may be attributed to the reduction of increased resistance in the ankle plantar flexors, thereby permitting more pronounced ankle dorsiflexion. This may also explain why the BRS improvement in the spasticity-with-contracture group was less marked than in the spasticity group. The improvement in lower limb FMA was less evident than that of BRS. A plausible explanation is that the patients in our study had relatively preserved motor function, with FMA scores around 20, which may have introduced a ceiling effect and contributed to the lack of statistical significance in the final results. Both groups showed improvement in BI scores. This improvement did not occur immediately following injection but emerged between weeks 4 and 12, suggesting that the amelioration of patients’ activities of daily living was not attributable to the immediate relief of increased muscle resistance, but rather progressed in parallel with the improvement in lower limb motor function. In other words, BoNT-A injections do not directly improve FMA scores or activities of daily living, and the co-occurrence of contractures likewise shows no substantial correspondence with either of these two aspects.

To our knowledge, this is the first retrospective study to directly compare the time course of BoNT-A efficacy between post-stroke patients with spasticity alone and those with co-existing spasticity and contracture using a multimodal assessment. While Gracies et al. previously demonstrated that severe soft tissue shortening impairs active functional goal attainment in a post-hoc analysis of the ULIS-II cohort, their study focused on the upper limb and did not examine the differential temporal dynamics of MAS, motor function, and ADL improvements (16). Our findings extend these observations to the lower limb and reveal a previously unreported dissociation between the time courses of resistance reduction, motor recovery, and functional independence. Consistent with Gracies et al., we found that the presence of contracture attenuates the magnitude of MAS improvement following BoNT-A injection (16). However, our study further demonstrates that this attenuation is evident as early as week 2 and persists throughout the 12-week observation period. Lindsay et al. demonstrated in a randomised controlled trial that early BoNT-A intervention (mean 18 days post-stroke) significantly reduces contracture development compared with delayed treatment; however, their design precluded direct comparison of established contracture versus spasticity alone, and they did not assess the temporal dissociation between spasticity reduction and functional gains (17). Falcone et al. (18), in a 14-year retrospective cohort, observed that chronic-phase patients required escalating BoNT-A doses, suggesting diminished treatment responsiveness over time, but they did not stratify outcomes by contracture status or report discrete time-point analyses. These findings are further contextualised by recent evidence that conventional rehabilitation, particularly muscle stretching, can mitigate non-neurogenic resistance and enhance BoNT-A long-term efficacy (19), and that multimodal approaches combining BoNT-A with adjunctive therapies such as extracorporeal shock wave therapy may be required for optimal management of contracture-plus-spasticity (20). The 2026 AHA/ASA scientific statement on post-stroke spasticity emphasises the necessity of distinguishing neurogenic from non-neurogenic contributions to increased resistance (3), underscoring the clinical relevance of our stratified approach. Future prospective studies with standardised rehabilitation protocols and longer follow-up periods are warranted to validate these retrospective observations and to optimise treatment algorithms for this challenging patient population.

This study has several limitations. First, the retrospective design precludes causal inference. Selection bias may exist in the allocation of patients to groups, and the observed associations may reflect confounding by disease chronicity, severity, or unmeasured factors. Additionally, baseline characteristics were not balanced between groups, as patients in the spasticity-with-contracture group had longer disease duration than those in the spasticity-only group, which likely confounded the observed treatment responses. The between-group difference in disease duration precludes attributing the delayed treatment response solely to contracture presence; chronicity itself may independently attenuate BoNT-A efficacy. Second, rehabilitation protocols were not standardised across the cohort, which may have further influenced functional outcomes. Third, the sample size was relatively small, limiting the generalisability of our findings. We did not perform multivariate adjustment due to sample size constraints; future prospective studies with larger cohorts are warranted to adjust for disease duration, baseline severity, and rehabilitation intensity. Fourth, the follow-up period was confined to 12 weeks; longer observation is required to ascertain the durability of BoNT-A effects in patients with established contracture. Finally, contracture severity was dichotomised rather than graded, potentially obscuring dose–response relationships. Future prospective, multicentre studies with standardised concomitant therapies, balanced baseline characteristics, and extended follow-up are warranted to validate these observations.

## Conclusion and future directions

BoNT-A markedly reduces post-stroke hyper-resistance and enhances motor function and activities of daily living; by contrast, concomitant contracture is associated with delayed and attenuated improvement in MAS and BRS.

Based on our findings, we recommend early BoNT-A injection for post-stroke spasticity to prevent contracture development and maximize therapeutic benefit. For post-stroke muscle hyper-resistance, a precise assessment prior to treatment appears necessary to guide subsequent therapeutic interventions, particularly botulinum toxin type A injections. Recently, we have also been exploring a multimodal assessment approach incorporating surface electromyography, isokinetic dynamometry, two-dimensional muscle ultrasound parameters, and elastography, in order to identify the pathological origins of muscle hyper-resistance. BoNT-A should be combined with complementary rehabilitation measures, including soft tissue stretching and joint mobilization, rather than used as monotherapy.

## Data Availability

The original contributions presented in the study are included in the article/supplementary material, further inquiries can be directed to the corresponding author.
